# Parents and nurses telling their stories: the perceived needs of parents caring for critically ill children at the Kilimanjaro Christian Medical Centre in Tanzania

**DOI:** 10.1186/s12912-019-0381-8

**Published:** 2019-11-13

**Authors:** Vivian Frank Saria, Lilian Teddy Mselle, Birgit Anne Siceloff

**Affiliations:** 1 Kilimanjaro Christian Medical Centre, PO Box 3010, Kilimanjaro, Tanzania; 2 Department of Clinical Nursing, Muhimbili University of Health and Allied Sciences, PO Box 65001, Dar es Salaam, Tanzania; 3 Weill Cornell College, 407, East 61st Street, New York, NY 10065 USA

**Keywords:** Critical care, Intensive care unit, Perceived needs, Parents, FGD, Tanzania

## Abstract

**Background:**

Parents have significant responsibility in the care of their critically ill children who have been admitted to the intensive care unit (ICU). When staying with their children in the hospital, they also have particular needs that should be adequately acknowledged and responded to by healthcare providers. Moreover, when their needs are not identified and addressed, parents may experience stress and anxiety as a result. This study describes the needs of parents caring for hospitalized critically ill children, as perceived by parents and nurses.

**Methods:**

This study used a descriptive qualitative research design. Five focus group discussions with nurses and parents of critically ill children, who were purposefully recruited, were conducted at the Kilimanjaro Christian Medical Centre Hospital. A qualitative content analysis guided the analysis of the data.

**Results:**

Two themes emerged from the perceptions of parents and nurses about the needs of parents caring for hospitalized critically ill children. These were: “engaging parents in the care of their children” and “receiving psychosocial support”. Both parents and nurses identified the importance of providing adequate information about their children’s progress, encouraging and involving parents in the care of their children and having flexible visiting time for parents was vital when caring for critically ill children.

**Conclusions:**

This study provides an in-depth understanding of parents’ needs when caring for critically ill children in the hospital setting. Nurses caring for these children should understand the needs of parents and integrate the parents into the daily care of their children. Nurses should also continuously support, inform and engage parents during child-caring procedures. Finally, visiting times for intensive care units should be flexible and allow more time for parents to connect with their hospitalized children.

## Background

The hospitalization of a child with critical illness can be overwhelming and stressful for parents. The experience of parents observing changes in their critically ill child’s condition has been compared to a ride on “an emotional rollercoaster” [1]. There are on-going difficulties as parents to also learn to adapt to developmental changes as their child matures - a situation that may be aggravated when parents are in a stressful environment such as an ICU and not well supported. Critical hospitalization is a situational crisis and parents might not have the ability to adjust to this extremely difficult situation. Parents will often focus on the immediate needs of their child, leaving their own needs unmet [2]. Parents often have significant responsibility in the care of their critically ill children when hospitalized. In some studies, parents reported that they expected to be involved in the care and receive continuous information about their children’s condition and prognosis [3]. On the contrary, most of the time nurses and other clinical staff attention is primarily focused on the needs of the critically ill children, with little consideration paid to the parents’ needs [4]. Parents require guidance from healthcare providers, especially when they are in high tech and potentially frightening environment like an ICU, in order to effectively provide care for their children [5, 6]. Parents rely on the guidance of nurses’ who are continually present in the ICU rather than other healthcare providers, including doctors, who commonly only meet with parents during ward rounds. Nurses are the healthcare providers most likely to provide emotional care to parents, in order to manage their emotional stress and other difficult situations [7].

Studies from other countries [8, 9] have reported that parents of critically ill children were present during certain procedures, stayed physically close to their children and were informed about their children’s condition by the healthcare team. However, in other studies, they were not included in the nursing care plans, and hospital visiting policy prohibited them from spending time with their children [5]. In Tanzania, only one person is allowed to be present with the child in the ICU. Other members of the family (and friends) are only allowed to visit during a 2-h visiting period so that they will not interfere with the child’s care, exhaust the child and potentially spread infection.

There is no body of literature exploring the needs of parents caring for critically ill children in the ICU from the perspective of parents and nurses in Tanzania. A few studies have focused on deficiencies in the care of seriously ill children, critical care services [10] and the satisfaction of family members of critically ill patients [11]. Similar studies have been conducted in developed countries [2, 3, 12], although the experiences and needs of parents of hospitalized critically ill children may be different from that of parents in developing countries, such as Tanzania, due to socio-economic and cultural differences. Because of limited resources, critically ill children in developing countries are commonly managed in general paediatric wards that are usually crowded with non-critically ill children. This study describes parents’ and nurses’ perceptions of the needs of parents, with a primary focus on their engagement in care and the psychosocial support they receive when caring for their critically ill hospitalized children.

## Methods

### Study design and setting

This descriptive qualitative study [13] explored the perception of parents and nurses caring for critically ill children. This study design was chosen to gain a deeper understanding of the needs of parents of critically ill children admitted to the hospital and to examine the differences in these experiences from studies conducted in other countries. The research was conducted between October and December 2016 at the Kilimanjaro Christian Medical Centre (KCMC) in Kilimanjaro region, Tanzania. KCMC is a tertiary and teaching hospital in Northern Tanzania with a 560-bed capacity, and also serves more than 11 million people at outpatient clinics. The hospital has 300 paediatric beds and admits about 1100 critically ill children annually [14]. The hospital was conveniently selected because of its accessibility to researchers. Specifically, this study was carried out at the paediatric burn, and surgical intensive care units, where critically ill children are admitted.

### Participants and recruitments

The purposive sampling strategy was used to recruit participants for the study [15]. The nurses in charge of the wards were asked to identify parents with critically ill children who met the inclusion criteria. All children who were admitted to the burn unit, paediatric ward and surgical intensive care unit considered being critically ill. The inclusion criteria were parents who had hospitalized children between 1 month and 12 years of age, who had been admitted for more than 76 h. It was assumed that 76 h of stay would be sufficient for the parents to realise their needs while in these units. In order to select a sample of nurses, the researcher approached nurses working in these units and administered a short screening questionnaire. The inclusion criteria were that nurses had to have worked in the unit for 2 years or more. The identified participants in both samples, parents and nurses, were then informed about the purpose of the study, issues of confidentiality and the voluntary nature of their participation. Those who agreed to participate in the study then provided written consent for their participation.

### Data collection procedure

Five (5) focus group discussions (FGDs) were conducted, 2 with nurses (*n* = 14), and 3 with parents (*n* = 24) of critically ill children. Each FGD had a minimum of six participants. Group discussion with nurses included both male and female participants. Before each FGD, written consent was obtained from participants by means of a consent form, which was written in Kiswahili. Kiswahili is the native language spoken by all the participants. To ensure privacy and confidentiality, the FGDs were conducted in the conference room adjacent to each ward and were moderated by the first author (VFS) in Kiswahili. The moderator was assisted by a trained research assistant who recorded the session using a digital voice-recorder and wrote any nonverbal cues observed from participants during the discussion. The research assistant had experience conducting health-related research and received a one-day training that primarily focused on the research objective and the process of data gathering. In order to guide the discussions, the moderator used the group discussion guides (Additional files 1 & 2) prepared by researchers and had a series of open-ended and probe questions regarding the needs of parents caring for critically ill children in the hospital. During the discussion, the nurses and parents were encouraged to actively participate. They were told that there were no right or wrong answers and therefore, should feel free to share their perceptions regarding parents’ needs while caring for their critically ill children. Each participant was given a unique number, and ground rules were set at the beginning of each discussion to ensure that each participant had the opportunity to share their perceptions. Furthermore, the moderator ensured that each participant had spoken during each discussion. Although achieving data saturation in focus group discussions is difficult [16], a saturation of data for group discussions with parents was reached after 5 FGDs, whereas the saturation of data in FGDs with nurses was not considered due to the limited number of nurses who were available to participate in the study during the data collection period. Data saturation is achieved when answers from the participants seemed to repeat information gained earlier and little new information is attained [17]**.** All discussions were audio-recorded after receiving verbal consent from the participants. Each group discussion session lasted between 40 and 60 min.

### Data analysis

The audio-recorded discussions were transcribed word for word and translated from Kiswahili to English by the first author (VFS), who is a critical care nurse, and the accuracy of the translations was cross-checked by the co-author (LTM). Translation of interviews from Kiswahili to the English language was essential to allow the non-Kiswahili speaking researcher (BAC) to engage in the analysis process. To check for accuracy of the translations, three transcripts were translated from the English version back into Kiswahili, and then a member of the research team compared the Kiswahili and English transcripts for differences and similarities while listening to the original audio-recorded discussions. After verification of accuracy in translation, the transcripts were saved on a password-protected computer. The qualitative content analysis guided the analysis process [18], with a focus on both manifest and latent messages in the data. This analytical framework was chosen because it is a concrete analytical framework that could be readily applied. The analysis began by reading transcripts and written notes from the FGDs several times in order to gain an understanding of the perceived needs of parents caring for critically ill children in the hospital. Emerging codes were identified. Text (meaning units) describing the needs of parents with critically ill children were extracted and condensed by shortening the original text while maintaining the core meaning. These condensed meaning units were further condensed into codes and then organised according to similarities and differences in order to form categories and themes. An example of the analysis is given in Table 1. Two researchers (VFS and LTM) independently analyzed the transcripts manually. Multiple coding was considered to be one of the methods to maintain rigour in qualitative research [19]. After the coding, common and emerging categories were discussed among members of the research team before group consensus was reached [13, 19]. The emerging categories were then compared between and within each FGD transcript.

**Table 1:** An example of the process of analysis

Meaning unit in the FGD text	Condensed meaning unit	Code
Every time I ask about the progress of my child they are just brushing you off, they give very brief information and not the detailed explanation. For four days I haven’t talked to the nurse, today she gave me a very short explanation that I couldn’t understand very well (...)’.	Not given adequate information and rarely discuss with nurses about the child’s progress	^a^ Not provided with adequate information about the child’s condition and prognosis

^a^This code was later included in the category ‘Being informed’

## Results

The 24 parents of critically ill children who participated in the focus group discussions described themselves as employed (*n* = 18), housewives (*n* = 4) or petty traders (*n* = 2). They had a median age of 54.5 years. Of the 14 nurses who participated in this study, 6 were from the paediatric ward, 4 were from the surgical ICU, and 4 were from the burn unit. All were registered nurses with working experience of 3 or more years (Table 2).

**Table 2:** Descriptive statistics of the participants’ characteristics

Nurses (*n* = 14)	Number	%
Age		
< 30	3	21
30–35	5	36
> 35	6	43
Ward		
Paediatric	6	43
Surgical ICU	4	29
Burn Unit	4	29
Education level		
Diploma	6	43
Bachelor degree	8	57
Working Experience (years)		
2–5	3	21
5–10	8	57
> 10	3	21
Parents (*n* = 24)		
Age		
20–35	18	75
> 35	6	25
Occupation		
Employed	18	75
Housewife	4	17
Petty traders	2	8

Two themes and four (4) categories emerged from the analysis of the perceptions of parents and nurses on the needs of parents when caring for hospitalized critically ill children (Table 3). The focus group discussion number is used to identify participants in the quotes.

**Table 3:** Themes and categories on the perceived needs of parents caring critically ill children in the hospital

No.	Themes	Categories
1	Engaging parents in care	Being informed
		Being part of the care
2	Receiving psychosocial support	Being encouraged
		Having a flexible visiting policy

### Engaging parents in care

#### Being informed

Timely and comprehensive information on the progress and prognosis of critically ill children admitted to intensive care from nurses was perceived to be of paramount importance by parents. Parents expressed that they were not given adequate information about the condition and prognosis of their children and that this was frustrating:

*‘Every time I ask about the progress of my child they are just brushing me off, they give very brief information and not the detailed explanation. For four days I haven't talked to the nurse, today she gave me a very short explanation that I couldn't understand very well … .* ’ **(**Parent, FGD #2**)**

Due to inflexible working styles, ICU staff were unable to provide adequate and timely information to parents regarding their critically ill children. However, parents often got information about their children from staff working elsewhere:

*‘Honestly, somebody who works in the hospital had eased getting information from the ICU nurses. I am sure without this person my communication and accessing surgeon in the ICU wouldn’t be as good as it is now*’ (Parent, FGD #1)

Nurses acknowledged the importance of providing adequate information to parents. However, they claimed that the shortage of staff was a barrier for them to provide adequate information:

*‘Most of the time parents are eager to know about their children’s condition, we usually provide them with information but not so comprehensive as we have many patients to look after so to spend adequate time with one mother when you have other children waiting for your care, it becomes difficult’* (Nurse, FGD #3).

*‘Parents with critically ill children always want to know about their Children’s prognosis. We provide only important information as it took a long time to describe in detail'*(Nurse, FGD #3)

It was further reported that parents often became part of the discussion about their children’s treatment plans and prognosis during major rounds. During this time, parents have the opportunity to ask questions and clear up any doubts or concerns they might have:

*‘During rounds, parents get to be involved and provided with adequate information about the progress of their sick children because during this time all ICU staff and other health providers taking care of children in the ICU discusses each child in detail’* (Nurse, FGD #5)

### Being apart of care

Parents of critically ill children are usually present with their children in the ICU and are commonly involved in direct patient care. This is very satisfying for them and increases their feeling of control over a difficult and stressful situation:

‘ … *in the paediatric ward, we usually used to bath our children and feeding them, even those who have feeding tubes we also feed them* ourselves’ (Parent, FGD #1)

But not all parents had the opportunity to be involved in caring procedures:

‘ *… if I get the opportunity to assist in the care of my child I will be very happy … ’* (Parent, FGD #4)

Nurses reported that parents’ participation in the care of their children is valuable. However, parents can become uncomfortable when certain medical procedures are carried out on their children in their presence and this can contribute to increased stress on their ill children:

*‘When we (nurses) conduct some procedures like suction or intubation to a child, you find that the parent screams a lot and therefore we ask them to stay away. ’* (Nurse, FGD #3)

Even though parents are happy to take part in the care of their critically ill children, they were concerned about the ICU environment and health care providers’ behaviours:

*‘To involve us, the ICU should be well-prepared and the healthcare providers should accept the practice, and allow us to participate in the care of our children’*(Parent, FGD #4)

On the nurses’ side, involving parents in their children care was seen as the solution to ease their workload:

‘ … w*ith the shortage of staff in the burn unit, we sometimes assign parents to soak their children’s wound before we clean and do dressing’* (Nurse, FGD # 3).

*‘We sometimes let parents do dressing to their children and feed their children so that they acquire skills of doing it when they go home*’ (Nurse, FGD #5)

### Receiving psychosocial support

#### Being encouraged

Parents have to be reassured about the care they provide to their children. Parents with critically ill children expressed that health care providers should be optimistic, use encouraging words and maintain a smiling face. A parent from the paediatric ward shared his opinion about the importance of nurses being positive and encouraging:

*‘As a second patient (parent of a critically ill child) we need care, hope, and encouragement from nurses and doctors. Sometimes the information that they give makes us tense and prevent us from sleep...you can give honest information with a bit of optimism. Don’t lie or deceive, as our children are critical but have to use suitable words. I think the nurses need the training to do this’* (Parent, FGD#1)

*‘ … my neighbour parent at ICU was very anxious, she needed to be supported and reassured that the machine that her son is using helps him to breathe properly, she needed to cope with her son’s critical situation’* (Parent, FGD#4)

Some parents turned to spiritual guidance as a way to find comfort, maintain hope, and practice patience and calmness:

‘*The illness is a test of patient's faith in God Almighty; the parents and their families should be always reminded of this thing to be more positive and hold onto hope’* (Parent, FGD#2)

‘*We [Muslims] say that all available treatments are only after Allah’s will; Allah is the ultimate healer’* (Parent, FGD#2)

Nurses also had this to share:

*‘ … we usually provide emotional support for those parents who are anxious and depressed’*(Nurse, FGD#5)

### Flexible visiting policy

Parents thought that family visits provided an opportunity for critically ill children to spend time with their loved ones, giving them a sense of love and belonging. In contrast, restricted visiting time decreased family interaction and feelings of support:

*‘ … truly an hour visiting time is not enough; the child needs his/her family and their close friends during recovery of the illness which is not facilitated with such restrictive visiting practices. Sometimes my son wants his father to stay with us a little longer, but unfortunately, this is not allowed here with such restricted hospital visiting policy****’*** (Parent, FGD #1)

*‘The visiting time was neither sufficient nor appropriate; we come from afar, sometimes we arrive towards the end of the visiting hour, we stay for only five to ten minutes then we are asked to leave’* (Parent, FGD#4)

*‘I prefer flexible, the least rigid visiting protocols as this will have a positive impact on the family and the child* … ’ (Parent, FGD#2)

Other parents thought that the presence of a visitors’ waiting room could be helpful:

*‘ … more inflexible visiting practices require a visitor’s waiting room with good furniture’* (Parent, FGD#2)

The hospital visiting policy however, allows visitors only 2 h in the afternoon; otherwise, only one parent is allowed to stay with the child:

*‘We allow only one parent to stay with a child; others have to come during visiting time this is according to hospital visiting policy’* (Nurse, FGD #3)

## Discussion

### Receiving information

Obtaining information from health care providers is the right of patients and their caregivers. Correct and timely information to patients and caretakers provides the necessary information to make informed decisions and contribute effectively to the care of their children [20]. Consistent with other studies [21–23], parents of critically ill children in this study expressed the need to be informed about their children’s care and procedures. Providing adequate and honest information is of great importance especially during the parents’ stay in the critical care wards. The parents of critically ill children admitted to the hospital should have access to information delivered in the way they could easily understand and their information needs should be discussed during each contact with a health professional. Parents should also be helped to understand the information, by being given the opportunity to ask questions, and be allowed to take part in the decision-making process around the care of their child, if possible. The ward rounds and visiting hours can be appropriate avenues for health care providers to share information with parents [24]. Further, continuous interaction of staff and parents during the process of providing care to the ill child is likely to make parents feel confident andmore comfortable to seek information [3]. During these times, parents should also be encouraged to ask questions, provided with timely and adequate information, and supported in their role as a critically important part of the healthcare team.

Because of the shortage of nurses, other members of the healthcare team, like social workers, should be allowed to provide information to parents in order to compliment the nurses’ role. This would ensure that parents of critically ill children are supported by all those involved in the care of their child. During training, social workers are taught how to provide information in an honest and empathetic manner [25] and are skilled at providing emotional support. They are trained to help patients and families understand a particular illness, help parents work through the difficult emotions surrounding a grave diagnosis and provide support when decisions need to be made about the care of their child. Nevertheless, nurses and doctors remain the key healthcare professionals responsible for supporting parents and other family members of critically ill children. They should demonstrate effective communication skills by continuously assessing parent needs and providing information in a timely and friendly manner. Other strategies used to support parents during their child’s hospitalization such as parent support groups [26], family conferences [27] and continuous staff-parent interactions during the process of care [3] are also important to ensure that parents share their feelings and get support. Miscommunication can lead to a prolonged stay, lack of continuity of care, suboptimal patient flow, readmission, patient dissatisfaction and increased parental stress and anxiety.

### Engagement into care processes

Parents’ involvement in their child’s care reduces anxiety and allows parents to feel supported and empowered, enhancing their coping mechanisms [3, 24]. Moreover, parents are critical to alleviating their child’s stress and improving their clinical outcomes. Bond et al. [28] reported that the involvement of parents in their child’s care increases their understanding of their child’s physical and emotional needs and prepares them for the caretaking role when the child is ready to be discharged. Parents commonly experience a sense of helplessness [3] when they do not know how to care for their children. Therefore, parents should be involved in the care of their hospitalized child, as has been reported in other studies in developed countries [3, 29].

While some parents in this study indicated an interest in being involved in their children scare, others were not comfortable taking part because they worried that they could cause harm to their children. Therefore, parents should not be forced to assume these responsibilities unless they are willing to do so. Nurses should assess parents’ need for involvement, information, proximity and support in each individual case. Also, nurse administrators should provide the resources necessary to organize and coordinate nursing care, in order to enhance a supportive clinical environment, which facilitates the engagement of parents in the care of their critically ill children.

### Flexible visiting policies

Parents in this study preferred an open visitation policy, which is also supported by the critical care group [30]. Restricted visitation policy is commonly practised in many intensive care units in the world, including Tanzania. As opposed to the opinion of participants in this study who wanted flexible visitation policy in which family and friends could see their critically ill children as often as they wanted, participants from other studies wanted some restricted and controlled visitation, allowing only family members to visit [31–33]. Further, consistent with the findings of this study, participants in a study by Olsen et al. [31] felt more supported when their families were present and wanted more flexible visitation. Taken together, these results highlight the importance of integrating patients’ and families perspectives when developing hospital visitation policies. Flexible visitation policy would foster parents and critically ill children’s emotional wellbeing by having close contact [34, 35] and not be isolated by restricted visiting practices. Flexible visitation is also one of the key strategies for the provision of patient-centred care [36]. Furthermore, helping parents meet these needs will enhance their well-being and coping abilities as they will be able to obtain information regarding their children’s condition and increase their satisfaction [25]. Other groups have also reported that both children and parents benefit emotionally from being close to each other [34, 35].

The benefits of open visitation have been reported, where patients feel supported [37] and safe [38], and families are more satisfied with care and less anxious [39]. With this policy, healthcare providers have more opportunities for communication and teaching [37]. Because of these benefits, open and unrestricted visitation in ICUs has been recommended by critical care groups [23]. The establishment of flexible visiting policies would promote an environment conducive to meeting the needs of parents and children [9]. From the findings of this study, even within an environment that provides unrestricted visitation, parents may not understand or process all information and, therefore, nurses need to provide continuous explanations, clarification and reinforcement to parents to minimize misconceptions related to their children’s care.

### Strength and limitations

The trustworthiness of the data was established using various methods including a purposive selection of participants and triangulation of data sources (i.e., parents, nurses) which provided depth and breadth of the perception of parents and nurses on the needs of parents caring for critically ill children in the hospital setting. Using an audio-recorder to record interviews, the Kiswahili language during data collection and a guided analytical framework for the analysis of the material from participants, the credibility of the findings was increased [40]. Furthermore, credibility was achieved through validation of the key themes via dialogue with all members of the research team as well as continuous evaluation of codes to ensure an accurate fit. Provision of direct quotes allows readers to judge the dependability of the findings. Based on the findings of this study, the perception of parents and nurses about the needs of parents with critically ill children in the hospital are relevant to the perceptions of parents and nurses in other countries that have a similar socio-economic context as Tanzania. Nevertheless, because the analysis of the FGDs was completed in English from translated transcripts, the quality of the participants’ accounts may be affected, as some Kiswahili words may not have a direct translation in English. However, the transcripts were verified by the research team fluent in Kiswahili and then back translated from English to Kiswahili to check the quality of translation and ensure that the translations were accurate. Further, all codes and themes were discussed among the researchers who were able to review the original transcripts.

## Conclusion

The engagements of parents in the care of their hospitalized child, sharing information from healthcare providers to parents, providing support and proximity of parents to their critically ill children were all identified as important needs of parents caring for critically ill children. To consistently integrate the needs of the parents of critically ill children into the care plan, nurses should identify the knowledge needs of both parents and children, and adequately support and provide care, thus increasing comfort and self-care. Nurses need to be proactive in engaging parents in the direct care of their children. This may facilitate adaptation to their children’s critical illness and the hospital setting. The government needs to consider implementing open visitation policies which support patient-centred care. This not only increases parents’ satisfaction with care but also increases the quality of that care. Research about how parents can be engaged actively with their child’s hospitalization in a way that is safe for patients is an important area to explore.

## Supplementary information

**Additional file 1:** Focus Group Discussion Guide for Nurses.

**Additional file 2:** Focus Group Discussion Guide for Parents.

## Data Availability

The data and materials are available from the corresponding author on reasonable request.

## Abbreviations

FGD: Focus group discussions
ICU: Intensive Care Unit
KCMC: Kilimanjaro Christian Medical Centre
