# Supplementary material for: Parents and nurses telling their stories: the perceived needs of parents caring for critically ill children at the Kilimanjaro Christian Medical Centre in Tanzania
Source: BMC Nurs. 2019 Nov 13;18:54. doi: 10.1186/s12912-019-0381-8 (PMC6854695; doi:10.1186/s12912-019-0381-8)
Supplement: Supplementary file 1 — Additional file 1: Focus Group Discussion Guide for Nurses. [file 12912_2019_381_MOESM1_ESM.docx]

**Focus Group Discussion Guide for Nurses**

1. What is your opinion about the needs of parents caring for critically ill children in your ward? (Probe: Physical, Emotional, Social?)
2. What information do you provide to parents of critically ill children in the ward? (Probe: progress of children, who provides the information, when do you give the information)
3. What is the responsibility of parents with critically ill children while in the ward? (Probe: how are they involved in care?)
4. How do the parents and families get to meet the critically ill children admitted in your ward? (Probe: opinion about visiting policy? adequacy of visiting time?)
