# Supplementary material for: Parents and nurses telling their stories: the perceived needs of parents caring for critically ill children at the Kilimanjaro Christian Medical Centre in Tanzania
Source: BMC Nurs. 2019 Nov 13;18:54. doi: 10.1186/s12912-019-0381-8 (PMC6854695; doi:10.1186/s12912-019-0381-8)
Supplement: Supplementary file 2 — Additional file 2: Focus Group Discussion Guide for Parents. [file 12912_2019_381_MOESM2_ESM.docx]

**Focus Group Discussion Guide for Parents**

1. What is your experience of caring for your child in the ward? (Probe: needs of parents when caring for their children; the support they receive from the health care provider)
2. What information do you receive from the health care providers while in the ward? (Probe**:** Child’s progress, nursing procedures, clarity of the information, opportunity to express concerns?)
3. How are you involved in the care of your children in the ward? (Probe: decision on the child’s care, caring process)
4. What support do you get from the health care providers while caring for your children? (Probe: Adequacy of visiting time)
5. What challenges do you face when caring for your children in the ward? (Probe: how did you deal with the challenges?)
